# Local responses to global sustainability agendas: learning from experimenting with the urban sustainable development goal in Cape Town

**DOI:** 10.1007/s11625-017-0500-y

**Published:** 2017-10-04

**Authors:** Zarina Patel, Saskia Greyling, David Simon, Helen Arfvidsson, Nishendra Moodley, Natasha Primo, Carol Wright

**Affiliations:** 1 Environmental and Geographical Science and African Centre for Cities, University of Cape Town, Cape Town, South Africa; 2 Mistra Urban Futures, Chalmers University of Technology, 412 96 Gothenburg, Sweden; 3 Independent Researcher, Gothenburg, Sweden; 4 Cities Support Programme, National Treasury, Pretoria, South Africa; 5 Organisational Policy and Planning Department, Directorate of the Mayor, Cape Town, South Africa

**Keywords:** Urban experimentation, Data and governance, SDG 11, Cape Town, Co-production

## Abstract

The success of the Sustainable Development Goal 11 (SDG 11) depends on the availability and accessibility of robust data, as well as the reconfiguration of governance systems that can catalyse urban transformation. Given the uneven success of the Millennium Development Goals, and the unprecedented inclusion of the urban in the SDG process, the feasibility of SDG 11 was assessed in advance of its ratification through a series of urban experiments. This paper focusses on Cape Town’s participation in piloting SDG 11, in order to explore the role of urban experimentation in highlighting the partnership arrangements necessary to allow cities to meet the data and governance challenges presented by the SDG 11. Specifically, we focus on the relationship between data and governance that lie at the heart of the SDG 11. The urban experiment demonstrates the highly complex and multi-level governance dynamics that shape the way urban experiments are initiated, executed and concluded. The implications of these dependencies illustrate that more attention needs to be paid at the global level to what data are important and how and where the data are generated if SDG 11 is to be met. Overall, this paper makes the case that the success of SDG 11 rests on effecting local level change and enabling real opportunities in cities.

**Electronic supplementary material:**

The online version of this article (doi:10.1007/s11625-017-0500-y) contains supplementary material, which is available to authorized users.

## Introduction

An expression of the gathering momentum for sustainable development is reflected in the recent adoption of the United Nations’ Agenda 2030 for Sustainable Development (United Nations 2015). This agenda has been enacted in part through the ratification and adoption of the Sustainable Development Goals (SDGs), which seek “to end poverty, protect the planet, and ensure prosperity for all” (United Nations 2016). Departing from their predecessors, the eight Millennium Development Goals (MDGs),^1^ the 17 SDGs apply to all countries. The SDGs seek to provide a more holistic and refined set of targets and indicators through which to measure progress towards sustainability over their lifetime (from 2015 to 2030). Fulfilling this agenda requires diverse innovative forms of active research engagement and support (Barnett and Parnell 2016; Satterthwaite 2016). This includes interdisciplinary collaborations between natural and social scientists and transdisciplinary team-building that brings together academic and non-academic (practice-based) researchers to investigate sustainability challenges of mutual interest.

Urban systems have received attention in the SDG process through Goal 11 (henceforth SDG 11), which seeks to ‘Make cities and human settlements inclusive, safe, resilient and sustainable’. This new agenda has embraced theoretical diversity through establishing coalitions of interests (Barnett and Parnell 2016). It acknowledges that responding to local contexts and stakeholder needs through engaging a diverse range of local partners has become standard rhetoric in fostering urban transitions (Marcotullio and McGranahan 2007; Parnell 2016). In translating this rhetoric into practice, this paper engages with what it means to engage with a range of partners to gain a practice based understanding of the new modes of governance that are necessary to foster urban transitions.

In this new sustainability agenda, bridging the divide between rhetoric and practice depends upon the ability of cities across the globe to report effectively on the targets and indicators of SDG 11 (See Table S1, Supplementary Electronic Material). The demand to actively measure progress, as captured through the different indicators of SDG 11 (and some relevant indicators in other goals), acts in turn as a trigger, requiring the active involvement of urban local authorities to make appropriate investments and interventions to achieve sustainability. The ability of local authorities to access, collate and report on targets and indicators depends on the relevance of the targets and indicators in a range of contexts, as well as the availability and accessibility of the data, which is often not solely within the ambit of local authorities. Similarly, the ability of city governments to utilise data to bring about urban change also depends on networks and governance arrangements at multiple levels.

Data and governance, therefore, sit at the core of the challenge posed by SDG 11. In other words, business-as-usual approaches that have either focussed on data^2^ or governance in isolation of each other need to change in order to be more responsive to the ‘wicked problems’ of enabling urban transitions (Evans et al. 2016).

The success of SDG 11 depends on the availability and accessibility of robust data, as well as the reconfiguration of governance systems that can catalyse urban transformation across the full spectrum of global urban contexts around the world (Satterthwaite 2016). However, these prerequisites are not a given, and the success of SDG 11 will be more challenging in urban contexts with weak governance and data management systems.

African urban local authorities generally still adhere to outdated structures and regulatory regimes, little changed since independence. These are commonly criticised for being inappropriate for present day needs, such as the integrated formulation and implementation of policies on sustainability and climate change (Stren and White 1989; Simon 1992), or to take advantage of new opportunities provided by information and communications technologies (Myers 2011). They remain bureaucratically hierarchical and unresponsive to demands for greater public accountability and citizen participation (e.g. Simon 1992; Dubresson and Jaglin 2002; Guèye 2004; Simone and Abouhani 2005; Myers 2011; Parnell 2016; Pieterse and Parnell 2014).

African cities, in particular, have notoriously weak governance systems and inconsistent data availability and quality (Pieterse and Parnell 2014). Data tend to be obsolete, incomplete and unreliable, despite many donor-funded initiatives to address these longstanding problems (e.g. through training and the provision of GIS and other equipment). The reasons for this are numerous and complex, defying simple solutions. Moreover, many economic activities and governance arrangements are undertaken informally (Huchzermeyer 2011), as demonstrated in Kisumu and Cape Town (Arfvidsson et al. 2016). In this context, operationalising SDG 11 effectively represents a major challenge in Africa.

Given the uneven success of the MDGs (Meth 2013), and the unprecedented inclusion of the urban in the SDGs, it was clear that the feasibility of SDG 11 needed to be assessed in advance of its ratification. As such, an urban experiment was set up to ground-truth the SDG 11 targets and indicators before they were finalised. A comparative international urban experiment was undertaken in cities across different global contexts during early 2015 to pilot the draft targets and indicators as they had been formulated at January 2015 (Simon et al. 2016).

Urban experiments such as the above are emerging within the social and applied sciences as a means of testing and addressing the challenges associated with implementing global agendas to create place-based policies (Evans et al. 2016; McFarlane 2011; Meth 2013). Following Bulkeley and Castán Broto (2013) p 363, we use the term ‘urban experiment’ to refer to “purposive interventions in which there is a more or less explicit attempt to innovate, learn or gain experience”. Urban experiments offer a means of introducing and testing new configurations in the translation of global initiatives into local contexts due to the potential they hold for catalysing learning and leveraging change (Bulkeley and Castán Broto 2013). Urban experiments have been applied in various contexts (Evans, 2016) to explore new interventions that address ‘wicked’ problems, such as sustainable development and climate change, or in the case of this paper, implemented through a pilot project that tested the feasibility of the draft targets and indicators comprising SDG 11.

In this paper, we focus on Cape Town’s participation in piloting SDG 11 in order to explore the role of urban experimentation in highlighting the partnership arrangements necessary to allow a city (the City of Cape Town (CCT) in this case) and a city’s local authority to meet the data and governance challenges presented by SDG 11. As our particular focus is on the relationship between data and governance that lies at the heart of SDG 11, we trace the local networks and relationships required to generate and access the data necessary to respond to SDG 11. In exploring the mutual but differentiated benefits of piloting SDG 11, the research outlined in this paper demonstrates commonalities across the global and local contexts in three areas: (a) better understanding of indicators and processes of securing data; (b) the development of new networks and partnerships that increase capacity for addressing the UN reporting requirements of SDG 11; and (c) the development of increased synergies to affect action and resource allocations.

## Literature review

### Defining urban experiments

Local authorities across the globe are increasingly documenting and advocating urban experimentation as a means of testing innovative tools for urban transformation through “real-world interventions” (Evans et al. 2016 p 2), particularly in the absence of widespread pre-existing local level transformation (see Bulkeley and Castán Broto 2013; Davison et al. 2016; Leck and Roberts 2015; Roberts 2008).

While urban experimentation can refer to initiatives to develop “technological innovations (designs, technologies, materials), social innovations (policy tools, financial mechanisms, changes to cultural norms) or both” (Castán Broto and Bulkeley 2013 p 94), in this paper we focus our attention on governance experimentation. In particular, we focus on the role that urban experiments play in building and expanding stakeholder networks, as well as the social learning that is enabled by this. The expanded networks that can emerge through experimentation have the potential to bring together diverse partners with different types of knowledge, something which might not be possible under ‘normal circumstances’.

These alternative partnership configurations are critical for learning and together generating new information that is credible, legitimate and salient (Cash et al. 2003 p 8086).^3^ Evans et al. (2016) link the importance of learning from real-world interventions with the broader emergence of reflexive governance, and the significance of learning within (and between) networks of urban actors, as posited by McFarlane (2011). Learning in turn results in enhanced capacity and appropriately targeted place-based interventions. In contrast to ‘best practice’ methodologies which imply top-down, expert-led interventions (see Patel et al. 2015), urban experiments can be seen as sites that open up “…relational spaces within organisations… [to allow] individuals or sub-groups within organisations to experiment, imitate, communicate, learn and reflect on their actions in ways that can surpass formal processes within policy and organisational settings” (Pelling et al. 2008 p 868).

Part of the appeal of urban experiments lies in the idea that experiments, by definition, do not always succeed. Posed as an ‘experiment’, an intervention with uncertain outcomes, but designed to induce change, can be allowed to run its course out of the limelight. Experimental projects are typically low risk, but have the potential to surface new insights that can yield high returns. Yet despite their low-key nature, urban experiments are significant for the social learning that takes place in the ‘shadow spaces’ of municipal institutions (Leck and Roberts 2015 p 61; Pelling et al. 2008). As a result of this learning, urban experimentation “potentially contributes to changes in norms, values, goals, operational procedures and actors that govern decision-making processes and actions” (Bos and Brown 2012 p 1341). In other words, they can prove to be valuable sources of (and stimuli for) innovation through locally appropriate, evidence-led policy and practice.^4^

Conditions that contribute towards the uptake of successful experimentation include (a) the timing of the intervention, (b) the topicality of the research locally and/or globally, (c) the existence of financial resources to fund the research, (d) the presence of champions to drive an experimental agenda and (e) a certain element of serendipity regarding the ability of stakeholders to partner (see Bos and Brown 2012 p 1350; Roberts 2008).^5^ Thus, urban experiments can provide new possibilities for engaging, both within institutions and in new configurations across institutions. Such new modes of engagement have the potential to affect practice in new ways and ultimately to support enhanced results and catalyse outcomes in the urban environment.

Despite the opportunities presented by urban experiments, Evans et al. (2016) highlight that urban experiments are not a panacea, nor are they inherently positive, as they too are riddled with politics (Evans 2016). These misgivings aside, the value of urban experiments is that they provide opportunities for introducing and testing new partnership configurations to create place-based policy responses to meet global and local imperatives.

### Localising the SDG 11

The increasingly multi-scalar nature of environmental policy agendas is also pertinent in the context of this study. Prompted by the Rio UN Conference on Environment and Development, there has been a shift since the 1990s from a predominantly global to a more local focus. This shift has seen the role of cities move increasingly (but not uncontestedly) to the fore. Hajer (2016) highlights the growing acknowledgement that cities are the “new agents of change on a global level”. Yet despite this change in scale, the growing relevance of, and extensive political support for, local interventions in environmental issues, the reasons for using the local as the site for action remain ambiguous “in terms of why the local is a pragmatic and/or theoretically sound scale for action, economy, and governance” (Lawhon and Patel 2013 p 1050). Urban experiments focussed on urban transitions (see Bulkeley 2010; Bulkeley and Castán Broto 2013) have demonstrated some of the challenges and shortcomings of the assumptions underpinning the invocation of the local to address global issues. Significantly, urban experiments corroborate the value of local governments working with local communities to solve local problems.

As the development of the SDGs was driven by the UN global environmental governance apparatus (and informed by the experience of the MDGs), they could appear to maintain a top-down approach for the monitoring and evaluation indicators for measuring (sustainable) development change in policy and practice. However, the 17 SDGs were developed between 2012 and late 2015 through a long process of widespread debate, negotiation and consultation not just among UN member states, but also non-state stakeholders, including international, national and sub-national organisations, professional associations, communities of interests and civil society bodies. Anchored by the Sustainable Development Solutions Network (SDSN), the body charged by the UN to produce the SDGs, the depth of consultation was unprecedented in UN processes. Despite the longstanding recognition from other stakeholders (including academics, analysts, practitioners) of the critical role of cities for delivering development outcomes (now and in the future), it is the first time that a mandatory UN statistical reporting mechanism includes a clear sub-national component in the promotion of sustainable development.^6^

Globally, local governments will be required to report on particular indicators in order to inform the national level’s responses to the international targets related to several of the SDGs. Of relevance to the successful implementation of SDG 11 was the lack of clarity regarding how an international reporting template can take into account local issues, when SDG reporting is co-ordinated at the national level. These debates amongst diverse stakeholders provided the opportunity for the Swedish-funded Research Centre, Mistra Urban Futures (MUF) to use its unique global research platforms (in both the global North and South) to undertake a pilot project to ‘ground truth’ the draft SDG targets and indicators in advance of their ratification as explained above (Simon et al. 2016).

## Methodology

The specific urban experiment explored in this paper is the MUF pilot project that tested the draft SDG targets and indicators in Cape Town on behalf of the SDSN prior to their ratification by the UN. While the ‘research template’ of the experiment was set by the Campaign for the Urban Goal, it was applied by local researchers in five diverse European, African and Asian cities; Gothenburg (in Sweden), Greater Manchester (in the United Kingdom), Cape Town (in South Africa), Kisumu (in Kenya) and Bangalore (in India). Together, the five cities “provide a reasonably representative sample of the diversity of urban contexts and conditions around the world… They possess very different local authority institutional capacities, and experience diverse levels of poverty, un- and underemployment, economic dynamism or stagnation, and social and environmental conditions” (Simon et al. 2016 p 50).

**Fig. 1:**
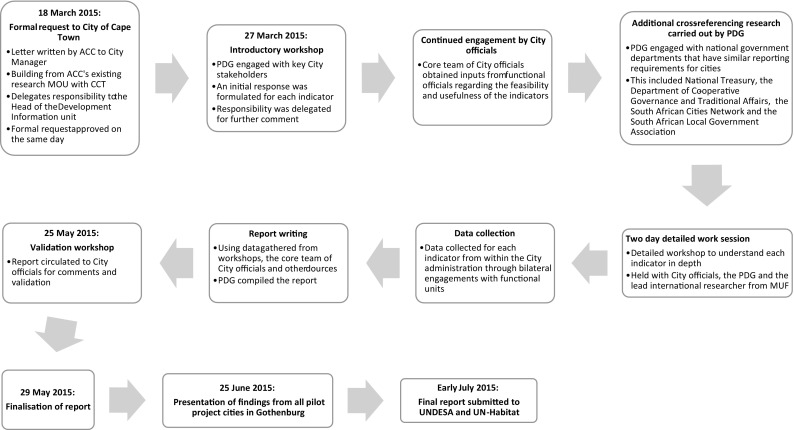
Time line of the Cape Town SDG 11 urban experiment

The pilot study sought to assess the feasibility of the primary and secondary indicators proposed to inform the targets that were set by the UN in order to achieve the SDG 11 (see Simon et al. 2016). In Cape Town, the data requirements for reporting on each indicator were tested against four parameters including (a) data availability, (b) data measurability, (c) data utility (i.e. usefulness), and (d) the custodianship of the data (i.e. where the data reside, who maintains them and who grants access to them). The process of assessing the data requirements for SDG 11 was conducted with local government officials across different municipal departments. The findings of this assessment were corroborated with other governance stakeholders, including national government, in order to provide a deeper understanding of the networks that are necessary to access the data necessary to report on SDG Goal 11’s targets and indicators (see Moodley 2015 and Fig. 1).

The authors of this article represent different stakeholder groups constituting a transdisciplinary research partnership. This partnership includes local government officials from the Development Information and GIS Department (DI Branch),^7^ local private sector consultants from Palmer Development Group (PDG), and academics locally from the African Centre for Cities (ACC, University of Cape Town) and internationally from MUF.

Participant observation and first-hand experience inform the documentation of the process of testing the SDG 11 indicators through the research partnership. Documentary evidence in the form of records from project meetings and the final project report, as well as thematic analysis of two presentations made by the project team to academic and City^8^ audiences also informed the findings related to partnerships. Key informant interviews with city officials informed the findings on local level learning. Through these multiple methods, we examine the conditions under which this urban experiment was undertaken.

## Observations and discussion

Our findings explore how (a) the urban experiment was initiated, (b) the importance of finding the correct ‘fit’ of partners to undertake the research, (c) what the different partners could offer to the collection and analysis of data**, and (d) the local and global changes (or impact) that resulted from the experiment. In the following sections, we reflect on these aspects of this urban experiment. Table 1 provides a summary of the findings in relation to the characteristics of urban experiments outlined in the literature review section above.

**Table 1:** Aspects and outcomes of the SDG 11 pilot urban experiment in Cape Town

Aspects of urban experiments		Aims/objectives and approach of the Cape Town SDG 11 Pilot Study	Outcomes of the Cape Town SDG 11 Pilot Study
Characteristics	Create place-based policies (Evans et al. 2016; McFarlane 2011; Meth 2013)	Increase the understanding of partnership arrangements necessary for reporting on SDG 11	Revised CCT’s 2016 business plan submissions based on understanding existing data gaps
	Introduce and test new configurations of partners (Bulkeley and Castán Broto 2013); inclusivity (Sengers et al. 2016)	Local knowledge partnership between ACC, PDG, and DI unit at CCT International knowledge partnership between ACC, MUF and SDSN Campaign for the Urban Goal	Developed new modes of governance through new networks within the CCT; and partnerships between the CCT and institutions generating city relevant data at the national scale that together increase capacity for addressing the reporting requirements
	Test assumptions and forge new pathways (Bulkeley 2010; Bulkeley and Castán Broto 2013; Evans et al. 2016)	Examine the potential for shifts from global rhetoric to local practice in terms of data and governance	Highlighted that Cape Town’s ability to report on SDG 11 depends on reconfiguring governance systems to deliver robust data
	Catalyse learning and leverage change through purposive interventions and lived experience (Bulkeley and Castán Broto 2013; Evans et al. 2016)	Understand data and governance needs to address SDG 11 through new spaces for engagement	The local–global project team and workshops that brought officials together in new configurations allowed for an enriched exchange of ideas
	Co-produce study problem and focus area	Broad parameters for the study were set by SDSN and the Campaign for the Urban Goal in partnership with MUF	Adjusted the global reporting template for SDG 11 to provide a better fit with the local context
	Introduce new technical innovations (Bulkeley and Castán Broto 2013)	Not an explicit goal of the Cape Town urban experiment	Adjusted the international reporting template for SDG 11 to obtain a more locally appropriate balance between output and input indicators Demonstrated the need for definitional clarity and the importance of considering the implications of informality in the articulation of SDG 11 indicators and targets
	Introduce new social innovations (Bulkeley and Castán Broto 2013)	Build and expand stakeholder networks Encourage social learning in order to catalyse change	Created links to national processes that rationalised city level indicators Obtained insights about the merits of transversal governance arrangements at the City through workshops with City Officials in configurations outside the rigid committee structures Expedited the research process through new partnership configurations
Features	Loosely configured and responsive (Cash et al. 2003; Murray Li 2007)	Adopt purpose-driven reporting templates and workshop configurations (rather than following specified predetermined structures)	Adjusted the international reporting template based on ‘ground-truthing’ in Cape Town Adjustments resulted in research that was ‘fit for purpose’ Allowed for feedback loops from the local to the global to break out of top-down patterns
	Experimentation as a ‘safe space’; contingency and uncertain outcomes (Farelly and Brown 2011; Karvonen and van Heur 2014)	Facilitate working with openness to context and without predetermined outcome expectations was through the appointment of PDG that was external to the existing MUF-ACC partnership The pilot project was not a City deliverable—therefore ‘success’ could be more loosely defined MUF’s approach to research co-production meant that this research was possible to be undertaken at short notice	Learning on data gaps in the Cape Town datasets, and sources of required data Datasets exchanges between departments through working in fluid workshop arrangements
	Out of the limelight	Incorporated into MUF MoU between CCT and ACC Allowed for new working configurations that went beyond the formal processes typical to local authorities	Study authorised through the City Manager’s Office due to its association with the existing MoU with the ACC Did not feature on City Official’s scorecards for reporting and performance purposes
Conditions for success	Timing (Bulkeley and Castan Broto 2013)	Ran from March-June 2015 Aligned with other programmes such as the City Urban Development Indicator Framework, ISO 37120 city indicators, and the transversal management shift within CCT	Delivery-focused departments did not want to lead the pilot as it was a short and intense study PDG were already working with National Treasury on city level indicators at the time. The strategic advantage of partnering on this global initiative gave the study salience Timing allowed for adjustments to the global SDG 11 before ratification in September 2015
	Topicality (Roux et al. 2017)	Development Information Unit is not delivery-oriented, but focused on data and worked in a transversal way across City departments PDG involvement was a good strategic fit with work rationalising indicators for the National Treasury	CCT gained a better understanding of indicators and processes of securing data
	Financial resources	Funded by MUF	Contribution of 85 h of unplanned and unallocated time from CCT personnel
	Champions and ‘fit’ of partners (Bos and Brown 2012)	Local: Development Information Unit at CCT. ACC as intermediary Global: MUF, Campaign for Urban Goal	Identification of the right partners within the local authority was important for the success of the pilot study Provided impetus for the DI unit’s approach to data acquisition, management and utilisation Impacted City structures through business plans informed by the findings of the pilot Influenced the final ratification of SDG 11
	Serendipity with partner arrangements (Roux et al. 2017)	Local: ACC and CCT history, and MoU with MUF Global: Links between Campaign for Urban Goal, MUF and UN	The appointment of PDG and leveraging the DI unit as City champions provided credibility to the study Diversity of knowledge bases and networks that informed the design and results of the pilot

## The initiation and execution of the Cape Town SDG 11 pilot

The opportunity to pilot the SDG 11 targets and indicators arose internationally due to the partnership between MUF and the SDSN. However, implementing locally a clearly defined research study over 3 months required the establishment of a research team drawing on partners from the local authority and beyond to cover the range of skills and networks. Given the urgent timeframes,^9^ establishing the City team for the research depended upon existing networks developed through the MUF-supported Knowledge Transfer Programme (KTP).^10^ However, the pilot project was not considered to be the right fit for the specific delivery-based City departments that participated in the KTP. These city departments resisted taking on a championing role for the project, as reporting on indicators through a range of other international and national processes was already placing strain on these departments. These departments were also hesitant to be the subject of yet another ‘pilot’ which would require major effort from within the City with little perceived grounding, feedback and benefit for the City. They expressed concerns that what appeared to be an ‘academically focussed initiative’ might outweigh the practical need and implementation imperatives of the City. Furthermore, concerns were raised about the level of engagement between departments that the project would entail in a short period of time, especially considering the absence of established institutional mechanisms for engagement. The need to invest time and resources in the SDG 11 pilot study in a resource-constrained environment, where the salience of the issue was not immediately apparent, meant that alternative entry points into the City were necessary.

Through the ACC’s wide-reaching networks within the City, the DI Branch was then approached to champion the project. The DI’s institutional mandate and focus on questions of data (rather than implementation), as well as their transversal^11^ positioning within the City, proved useful to overcome some of the concerns raised by the other departments initially approached by the ACC. The ACC’s partnership with MUF, and the credibility they have built with the City, provided the momentum for the establishing a new knowledge network, and the DI Branch believed that “[t]he opportunity to partner and collaborate is always an opportunity to learn” (Manager: DI Branch, presentation on 3 August 2015).

Establishing a research team to partner with the City on this high-profile, short-term project was not straightforward. Time constraints and already determined work agendas for the year meant that ACC researchers did not have the capacity to conduct the research and instead played the role of intermediary. PDG was strategically contracted as the research leads as they were deeply embedded in developing national, provincial and municipal systems for monitoring and evaluating municipal performance over the 15 years of the democratic system of local government. At the time of the study, they were working on a detailed programme for the National Treasury, through the Cities Support Programme (CSP), to rationalise city-level indicators for increasing the efficiency and effectiveness of reporting in relation to delivery and outcomes. The strategic fit between the piloting of SDG 11 and the indicator rationalisation effort of the national government was immediately apparent to the PDG team.

Whilst establishing the knowledge network and partnerships was one aspect of initiating the experiment, obtaining permission to conduct the project at the City was also necessary. Ordinarily, a project such as this one would not have been possible to initiate, given the requirements of the City’s rigid supply chain management, budgeting and work stream management procedures. However, building the project into the established Memorandum of Understanding between the ACC and the CCT on the existing MUF-supported Programme allowed the ACC to seek the support of the City Manager for the SDG 11 pilot research project and to request him to delegate responsibilities to the Manager of the DI Branch. Once the letter was approved (the very same day it was submitted, indicating a dedication to the project from the City team involved in the process), the research could begin.

The timing of the study was serendipitous for CCT. Momentum around indicator and monitoring and evaluation related projects and processes (e.g. The City Urban Development Indicator Framework) and the beginning of transversal management work was gathering during 2014, which formed a solid base to build on and extend through the pilot project. Additionally, the pilot project offered an opportunity to the City to improve professional practice in terms of enhanced capacity, and access to, new and expanded networks, resources and tools, all of which “would hold [them] in good stead” (Head: Policy and Research, DI Branch, presentation on 3 August 2015). This was particularly appealing because the City is “going to be asked to do a lot more with less, and this was one project with multiple outcomes” (Manager: DI Branch, presentation on 3 August 2015). The CCT team, already working with indicators, were exposed to a high-profile project which built on and enhanced the exposure of the City to the pre-existing global–local MUF-ACC MOU and partnership.

The conditions for the initiation of the urban experiment then were a combination of opportunities posed at the international and local level. Establishing the knowledge network to conduct the project was crucial to the successful undertaking and completion of this experiment. The input and commitment from the City officials was significant, as City officials alone spent 85 h on the collation of data, a task that was over and above mandated work plans. This indicates the extent to which complex challenges presented by global projects such as that of SDG 11 require the capacities of numerous stakeholders (Polk and Kain 2015). The local level opportunities discussed here emphasise the importance of timing, credibility and salience for implementing global projects (Cash et al. 2003).

## Data and partnerships

The assessment of the existing, available and required data to report on the targets and indicators necessitated partnership arrangements at three levels: (a) within the CCT, new partnership arrangements between departments were necessary; (b) local partnerships between the CCT, PDG and the ACC were required to co-design the research instrument to ensure local relevance; and (c) the global partnership between ACC and MUF provided an opportunity to link local engagement with global agenda setting.

Institutionally, the experiment was implemented through the CCT administration, which was necessary for accessing, processing and incorporating data and information from within the organisation. The core team of City officials engaged with respective functional officials to obtain feedback about the feasibility and usefulness of the indicators (see Fig. 1). Detailed work sessions on the study were held, unconfined by the ‘normal’ committee structures that determine inter-departmental engagements. This allowed the City line officials and the research team to reflect on the indicators in the context of the City’s own indicator processes. Work sessions included a transdisciplinary mix of City officials across line departments, the PDG team, an academic from the ACC, and in one instance, the lead international researcher from MUF for a global perspective. Following the consolidation of the research, local ‘ground truthing’ was sought by circulating the report to City officials for their feedback. These comments were incorporated into the final report that received validation globally through its presentation to the representatives of the pilot project teams of the other participating cities’ at a workshop in Gothenburg in June 2015.

Social learning at the local level emerged as the research team engaged closely with City departments at a detailed level on the current indicators and data, as well as current gaps and possible alternatives. The workshop spaces developed through the project (Fig. 1) brought officials together in new configurations, thus facilitating the development of new knowledge networks. Furthermore, the relational space created by the pilot project catalysed a proactive approach to respond to new data requirements (see Pelling et al. 2008). This allowed officials from the DI Branch to surpass formal procedures in order to respond to the pilot project’s requirements, an essential aspect of which was partnering with PDG in order to facilitate the collation of data beyond the scope of local government. As this research was undertaken in an experimental space, it allowed officials to operate more flexibly than is usually possible within the confines of bureaucratic procedures. These new mechanisms for cross-departmental engagement allowed City departments to apply their learning to other projects and to develop tools and processes for managing data to short deadlines.

Whilst partnerships were important for the initiation and facilitation of the experiment, they were also significant for the credibility of the results and the collection of robust and reliable data to inform the final report. As a team, the CCT, PDG and ACC were able to develop an open and iterative process of co-conceptualising the methods, tools and means of analysis to inform the framework, which was grounded in the SDG 11 brief and objectives. A further benefit of this diverse partnership was the opportunity to draw on PDG’s experience in evaluating and monitoring the performance of government institutions. This informed the methods to ground the findings in ways that better reflected the local context. The PDG undertook additional research to cross-reference the proposed SDG11 indicators with other local indicator programmes across a range of national government departments (see Fig. 1). Potential synergies with other processes included (a) the CSP’s development of a set of outcome indicators for the built environment functions of cities in South Africa developed by the CSP, and their process of rationalising and reforming the reporting burden placed on local governments; and (b) the ISO 37120 city indicators, arising from the Global City Indicators Facility, which were established to measure service delivery and the quality of life in cities globally.^12^

In identifying the sources of data required for reporting, it was clear that stakeholder networks and sources beyond the City had to be accessed. For example, data sources such as the nationally collected General Household Survey were found to be either relatively weak or underutilised for SDG 11 reporting, although the data could be useful to local governments. It was noted by participating officials that cities need to be better engaged with Stats SA about the usefulness of underutilised instruments like the General Household Survey in being able to provide good data on a regular basis for the planning and performance monitoring requirements of municipalities. As a result, there is significant scope for national and international investment in systems that would be shared at the city level to collect, warehouse, manage, report on, share lessons and knowledge from those sets of data.

The project team’s engagement with gaps in indicator data laid the foundation for more formal future indicator processes. The institutional value of working in reconfigured spaces was reinforced by a member of the team when she added that the evidence-based and interdisciplinary nature of the research, “support[ed] the City’s transversal work on urban complexities” (Head: Policy and Research, DI Branch, presentation on 3 August 2015). Working in a way that sought to overcome the “boundaries of the current compartmentalization of policy-making” (Polk and Kain 2015: 2), the pilot project helped the department to access information from different sources in the City and to understand the related data issues. This in turn improved the DI Branch’s understanding of (a) the different complex urban and institutional challenges, (b) the experiments underway to find alternative solutions and (c) the solutions that are being implemented in various departments.

The experiment provided the opportunity for the CCT to begin collating the many datasets that exist across the municipality that in many cases are produced and utilised within individual departments but are not known or readily available to other departments. Furthermore, key data gaps were identified,^13^ which have directly informed the CCT’s future work plans. CCT’s internal learning was around deepening the understanding of the City’s existing indicator data processes, systems and capacity. This learning then directly influenced the better understanding of what was needed for the pilot and future indicator work within the City. The DI Branch is currently driving processes to continue to develop their capacity around urban development outcome indicators. This is in alignment with broader work on monitoring and evaluating, outcomes and impact, as well as on ensuring the achievement of *real* change and benefits within the City. Officials were able to source datasets held by different departments “despite some competitiveness and protective behaviour” over the data sets (Moodley 2015: 70), undertake initial quality control and to start considering how to bring these together in a central, useful space so that a wider range of practitioners have access to them. City practitioners noted too that there is an increasing awareness within the City of the need for effective monitoring and evaluation. This has been indicated through a range of City departments requesting assistance from the DI Branch in developing indicators and offering assistance with monitoring and evaluation processes. This included the City’s work on Built Environment indicators to support its Built Environment Performance Plan.

For the City, links with organisations generating data beyond the City have been enhanced through engaging with PDG’s networks. These include links through PDG’s work with the National Treasury on rationalising indicator reporting requirements emanating from national government departments (and specifically the National Treasury) and drawing on the PDG’s historical body of work on local government indicators. In addition to learning about the data requirements for future reporting, numerous immediate outcomes locally emerged from the experiment. For example, officials from the City’s DI Branch indicated that they integrated some of this work into their 2016 business plan, and that they have started further research and background investigation around some of the areas where the City felt more refinement would be necessary. Some of these investigations include looking at indicator processes elsewhere in the world, focusing particularly on other cities.

For PDG, this research presented an opportunity to enrich its body of local government indicator work. Up to the point of this urban experiment, PDGs scope of work on indicators was undertaken entirely domestically. This experiment allowed exposure to global and multi-lateral perspectives.

For the ACC, some of the benefits that accrued locally included diversifying partnerships within the City, as well as facilitating new local partnerships between CCT and PDG to generate deeper research on City processes. Furthermore, direct input into the UN SDG process enhances the ACC’s global influence and contribution. Globally, the evaluation framework that was co-developed by the project team was applauded at the June 2015 Gothenburg meeting. All participating cities were required to adopt the Cape Town framework.

## Learning from the local as a site of global action

The pilot provided the opportunity to probe some of the concerns about using the local scale as a site for global action. Unlike other UN processes such as the MDGs, the pilot made provision for a feedback loop from the local to the global. Table S1 in Supplementary Electronic Material documents the extent to which changes to the targets and indicators resulted from the urban experiment conducted in Cape Town and the other cities. The changes in the text of the targets and indicators prior to ratification demonstrate the potential of urban experimentation to break from the usual top-down approaches related to the monitoring and evaluation of indicators. While the pilot was not labelled as a ‘best practice’ approach to monitoring and evaluation, the SDG's focus on the local scale does in some way acknowledge the need for change in how development is evaluated by international agencies.

The significance of what the local can offer to global reporting was apparent even during the data collection phase. Following the terms of reference provided by SDSN, PDG created a reporting template that sought to examine each indicator according to its availability, measurability, utility and custodianship. After applying these categories to the different indicators, it became clear that it was not feasible in the Cape Town context to report on all four aspects. The categories were then adjusted to limit the research to dimensions of feasibility and usefulness of the data (see Simon et al. (2016) for details). Using the feasibility and usefulness criteria contained in the assessment framework, it was found that most of the pilot project’s indicators are useful to collect and would be increasingly measurable with greater refinement, systems development and collaboration between cities and national stakeholders.^14^ This example illustrates how urban experimentation allows for increased responsiveness to contextual issues (Murray Li 2007 cited in Bulkeley and Castán Broto 2013). In this case this meant that the research questions across the SDG 11 pilot could be brought into better alignment with the other partners and needs, in order to ensure that the research is fit for purpose and context.

Some further data challenges and contradictions that emerged during the experiment with can have significance for global learning. These relate to the challenges of addressing informality through the proposed targets and indicators of SDG 11, as detailed by Simon et al. (2016). For example, in the context of informality that characterises much economic and human settlement activity in Cape Town (and also in Kisumu and Bangalore among the five participating cities) there is a real need for appropriate data collection and accounting systems.^15^ The Cape Town experiment highlighted both the challenges related to the co-existence of formal and informal urban settlements and identified how best to access, manage and analyse the relevant data to present an integrated view of the city. This may require the use of Geographic Information System (GIS) tools and spatial modelling to provide a deeper insight into human settlement trends, economic activity and transport. Indicator sets addressing ‘wicked’ problems thus need to go beyond performance management. Indicator sets need to constitute useful tools for sustainable city planning to increase their relevance for to stakeholders who can use them to inform decisions on a local level. In such an approach indicators are meant to complement one another to present a more holistic picture rather than simply act as stand-alone management tools. To achieve this, individual indicators must be allowed to compromise with certain criteria that mainly have measurability as their purpose, as we design indicator sets that work as a whole. If indicator sets are designed and implemented for reporting as a coherent whole, they also require dialogue and participation by a range of actors in integrated planning.

A further challenge was the alignment of the proposed indicators with those that already exist within the CCT. In collecting information on data feasibility and usefulness, officials found that the wording of the proposed SDG 11 indicators had to be analysed in fine detail. In doing this, it became apparent that a number of definitional issues would need to be clarified globally before the indicators could be employed by cities to inform each target. The ambiguity contained in the terminology used in the targets raised questions in the Cape Town context including the following: (a) whether to measure in units of population or household, (b) how to define the urban edge, (c) how to understanding urban agglomeration, (d) what is meant by terms such as ‘city’, ‘built-up area’, ‘public space’, ‘green space’, and ‘open space’, among others. Given these definitional issues, the officials who participated in the study articulated the need for a coherent indicator framework that includes the involvement of municipal practitioners early in the international negotiation exercise, so that they could ‘ground truth’ the indicators in local contexts.^16^ Overall, Cape Town’s experience of experimenting with SDG 11 provided an opportunity for the City to gain “a better understanding of the data and how we can respond” (Manager: DI Branch, presentation on 3 August 2015).

Working through these questions of definitional ambiguity has benefitted the global SDG 11 initiative because it has demonstrated the local variations and hence complexities involved in any comparative exercise of this nature. At the same time, at the local level, the officials who participated in the Cape Town urban experiment have emerged with a deeper appreciation of what it takes to work in the indicator space, indicating that social learning was facilitated through the experiment. The pilot was appreciated as a unique opportunity to make a contribution to the testing of the draft targets and indicators for a global process. Furthermore, through this process, the awareness and knowledge of the mandates of the other departments within the city government were deepened. Similarly, municipal practitioners were also afforded a greater understanding of what sits in sectoral indicators and, accordingly, where the data challenge lies. For example, the SDG 11 pilot project raised awareness of the role (and importance) of cross-sectoral indicators when working on complex urban challenges. It also confirmed that there are more input and output indicators than there are indicators that measure outcomes and longer-term impacts.

## Conclusion: learning from experimenting

Piloting SDG 11 in Cape Town provided an opportunity to experiment with the feasibility of the data and governance requirements embedded within SDG 11 prior to its ratification. The findings from experimenting with SDG 11 in Cape Town illustrated both the complex intra-local and multi-level governance arrangements that underpin the acquisition of data, thereby suggesting the extent to which governance arrangements need to be reconfigured and supported to foster urban change.

The study makes a case for the importance of urban experimentation for learning and leveraging change in urban contexts. The results of this experiment contributed to significant revisions in the final targets and indicators adopted by the UN General Assembly in September 2015. The changes in the text of the targets and indicators prior to ratification demonstrate the potential of urban experimentation to break from the usual top-down approaches related to the monitoring and evaluation of indicators. The feedback loops built into the mechanisms to report back the findings of the Cape Town urban experiment to a global audience ensured that local ‘ground truthing’ formed the basis of the global SDG 11 framework. Furthermore, the reconfigured agenda setting and prioritisation processes within the City of Cape Town, resulting from the pilot study, are evidence of the significant learning that emerged at the local level. The flexibility of urban experiments had an important role to play in allowing the study to be responsive to local conditions. The impact and value of experimentation for ground-truthing and informing local and global policy initiatives were valorised through this urban experiment.

Whilst the experiment has already been valuable in shaping local and global processes, the extent to which this value will have enduring effects for cities across the globe is dependent on the acknowledgement of the importance of invoking complex knowledge networks to report effectively on SDG11. Such networks must be acknowledged and supported to enable cities to learn and leverage change.

## Electronic supplementary material

Below is the link to the electronic supplementary material.

Supplementary material 1 (DOCX 24 kb)

## Footnotes

1 The unintended consequences for urban informal settlements that arose through the translation of global aspirations at the local level in the implementation of the MDGs are detailed in a poignant paper by Meth (2013). The cautions raised in this paper provide a rationale for the need to ensure that similar disjunctures are avoided in the adoption of the SDGs.

2 In a discussion on the politics of experimentation, Evans et al. (2016) caution against a data driven approach to urban governance.

3 Cash et al. (2003) p 8086 assert that boundary work, like urban experiments that brings together different stakeholders, “highlights the prevalence of different norms and expectations in the two communities regarding such crucial concepts as what constitutes reliable evidence, convincing argument, procedural fairness, and appropriate characterisation of uncertainty”. These ‘crucial concepts’, as well as other, less tangible factors that influence decision-making, are underpinned by tacit knowledge that is necessary to surface in order to catalyse change in institutions that are bound by bureaucratic management styles (see also Owens 2005).

4 While useful, the language of experimentation should be used carefully, as the notion of an experiment suggests laboratory-like conditions. Voß and Bornemann (2011) p 21 caution that learning “does not take place inside a scientific laboratory, somehow detached from immediate stakes and interests of actors, but in the real world… Consequently, the framing, observation, and interpretation of sustainability experiments are highly reflexive exercises” (2011) p 21. Bos and Brown (2012) p 1341 concur.

5 Bulkeley and Castán Broto (2013) p 367 note that in some cases, “[r]ather than creating protected spaces through which innovation can be fostered and system change developed, experiments could provide grist in the urban mill, creating conflict, sparking controversy, offering the basis for contested new regimes of practice”.

6 The Campaign for the Urban Goal played a significant political role in driving this significant scalar shift. See http://www.urbansdg.org.

7 The Development Information Branch is part of the Development Information and GIS Department, Corporate Services Directorate. With the restructuring of the municipality in 2016, this Branch has moved into the Directorate of the Mayor and into a new Department, now called the Research Branch, Organisational Policy and Planning Department, Directorate of the Mayor, City of Cape Town.

8 CCT refers to the metropolitan municipality and its governance and institutional structures. ‘City’ is used interchangeably with CCT.

9 Project initiation in March 2015 with delivery from all pilot cities by June 2015, in advance of the September ratification left a research window of 3 months.

10 The KTP, which ran between 2012 and 2015, sought to make policy for sustainable development more legible and defensible by fostering different spaces for engagement for City officials and other stakeholders (see Greyling et al. 2016).

11 The City of Cape Town’s transversal management system (TMS) complements the existing hierarchical organisational structure with platforms for communication and decision-making across directorates/departments and supports the change of the organisational culture from a ‘silo’ to a more integrated and coordinated system. In an increasingly complex urban environment, many of the challenges the City aims to address require collaboration among multiple departments. The TMS is a management device used to ensure that the City’s directorates collaborate to improve integration and coordination of service delivery and planning by creating structures and processes in which political leadership, senior managers and officials can communicate and work together. At the time of the study, the TMS was in its infancy, without structures for implementation.

12 At the time of research, members of the CCT team were, through a separate internal project, reviewing the City’s readiness to complete the ISO process. At the time of writing, CCT has become a member of the World Council for City Data (WCCD) and is in the final stages of the ISO certification process.

13 City indicators that needed development or refinement included: disaster risk management, open/green space, social development and cultural heritage.

14 A project scoping document was prepared and is being used as a guide for the Data Technical Working Group, and the City Support Programme, National Treasury to engage with StatsSA and the metros on how the National Statistical System can better support metros to transform their cities, by having the most appropriate data for city planning, monitoring, evaluation and reporting. The UN World Data Forum held in January 2017, provided some examples of how other countries and cities are strengthening their data capability. The project and process is ongoing with the most recent meeting of the Data Technical Working Group in May 2017 providing the opportunity for metros to share their data needs for city spatial transformation and reporting on SDGs, the interfaces with StatsSA, and to understand the metros’ statistical and strategic data capabilities.

15 For example, the context of informality that characterises much economic and human settlement activity in Cape Town and also in Kisumu and Bangalore (Arfvidsson et al. 2016) requires appropriate data collection systems and accounting systems.

16 For details of this and other project findings, see Simon et al. (2016).
