# Supplementary material for: Local responses to global sustainability agendas: learning from experimenting with the urban sustainable development goal in Cape Town
Source: Sustain Sci. 2017 Oct 4;12(5):785–97. doi: 10.1007/s11625-017-0500-y (PMC6086247; doi:10.1007/s11625-017-0500-y)
Supplement: Supplementary file 1 — Supplementary material 1 (DOCX 24 kb) [file 11625_2017_500_MOESM1_ESM.docx]

| **Table S1: Targets and indicators of SDG 11 prior to ratification** | | | |
| --- | --- | --- | --- |
| **Draft target/indicator Jan 2015 where different from final** | **Final target Sept 2015** | **Final indicator Sept 2015** | **Change from draft** |
|  |  |  |  |
| **Indicator 11.1.1** Percentage of urban population living in slums or informal settlements  **Indicator 11.1.2** Proportion of population that spends more than 30% of its income on accommodation | 11.1 By 2030, ensure access for all to adequate, safe and affordable housing and basic services and upgrade slums | 11.1.1 Proportion of urban population living in slums, informal settlements or inadequate housing | **Indicator 11.1.1** - ‘or inadequate housing’ added.  **Indicator 11.1.2** - deleted |
| **Indicator 11.2.1** Percentage of people living within 0.5 km of public transit [running at least every 20 minutes] in cities with more than 500,000 inhabitants  **Indicator 11.2.2** Km of high capacity (BRT, light rail, metro) public transport per person for cities with more than 500,000 inhabitants | 11.2 By 2030, provide access to safe, affordable, accessible and sustainable transport systems for all, improving road safety, notably by expanding public transport, with special attention to the needs of those in vulnerable situations, women, children, persons with disabilities and older persons | 11.2.1 Proportion of population that has convenient access to public transport, by sex, age and persons with disabilities | **Indicator 11.2.1** - made more relative and qualitative (‘convenient access’) instead of arbitrary thresholds.  **Indicator 11.2.2** - deleted |
| **Indicator 11.3.1** Ratio of land consumption rate to population growth rate at comparable scale    **Indicator 11.3.2** Cities with more than 100,000 inhabitants that implement urban and regional development plans integrating population projections and resource needs | 11.3 By 2030, enhance inclusive and sustainable urbanization and capacity for participatory, integrated and sustainable human settlement planning and management in all countries | 11.3.1 Ratio of land consumption rate to population growth rate  11.3.2 Proportion of cities with a direct participation structure of civil society in urban planning and management that operate regularly and democratically | **Indicator 11.3.1** - ‘at comparable scale’ deleted  **Indicator 11.3.2** - city size threshold replaced with proportion of all cities |
| **Indicator 11.4.1** Percentage of budget provided for maintaining cultural and natural heritage  **Indicator 11.4.2** Percentage of urban area and percentage of historical/cultural sites accorded protected status | 11.4 Strengthen efforts to protect and safeguard the world’s cultural and natural heritage | 11.4.1 Total expenditure (public and private) per capita spent on the preservation, protection and conservation of all cultural and natural heritage, by type of heritage (cultural, natural, mixed and World Heritage Centre designation), level of government (national, regional and local/municipal), type of expenditure (operating expenditure/investment) and type of private funding (donations in kind, private non-profit sector and sponsorship) | **Indicator 11.4.1** Yes - more detail and budget replaced by actual expenditure  **Indicator 11.4.2** - deleted |
| **Target 11.5** By 2030, significantly reduce the number of deaths and the number of people affected and decrease by [x] per cent the economic losses relative to gross domestic product caused by disasters, including water-related disasters, with a focus on protecting the poor and people in vulnerable situations  **Indicator 11.5.1** Number of people killed, injured, displaced, evacuated, relocated or otherwise affected by disasters  **Indicator 11.5.2** Number of housing units damaged and destroyed | 11.5 By 2030, significantly reduce the number of deaths and the number of people affected and substantially decrease the direct economic losses relative to global gross domestic product caused by disasters, including water-related disasters, with a focus on protecting the poor and people in vulnerable situations | 11.5.1 Number of deaths, missing persons and persons affected by disaster per 100,000 people  11.5.2 Direct disaster economic loss in relation to global GDP, including disaster damage to critical infrastructure and disruption of basic services | **Target 11.5** - percentage replaced by ‘substantially’  **Indicator 11.5.1** - removed injured, relocated and evacuated persons, and ratio per 100k people added  **Indicator 11.5.2** – reframed and broadened from housing units to total direct economic loss |
| **Indicator 11.6.1** Percentage of urban solid waste regularly collected and recycled (disaggregated by E-waste and non-E-waste)  **Indicator 11.6.2** Level of ambient particulate matter (PM 10 and PM 2.5) | 11.6 By 2030, reduce the adverse per capita environmental impact of cities, including by paying special attention to air quality and municipal and other waste management | 11.6.1 Proportion of urban solid waste regularly collected and with adequate final discharge out of total urban solid waste generated, by cities  11.6.2 Annual mean levels of fine particulate matter (e.g. PM2.5 and PM10) in cities (population weighted) | **Indicator 11.6.1** Reworded to emphasise adequacy of final discharge of all categories  **Indicator 11.6.2** Reworded for clarity and to introduce population weighting |
| **Indicator 11.7.1** Area of public space as a proportion of total city space  **Indicator 11.7.2** Proportion of residents within 0.5 km of accessible green and public space | 11.7 By 2030, provide universal access to safe, inclusive and accessible, green and public spaces, in particular for women and children, older persons and persons with disabilities | 11.7.1 Average share of the built-up area of cities that is open space for public use for all, by sex, age and persons with disabilities  11.7.2 Proportion of persons who are victim of physical or sexual harassment, by sex, age, disability status and place of occurrence, in the previous 12 months | **Indicator 11.7.1** - refined to specify average share and make explicit that all key categories of population should be included  **Indicator 11.7.2** totally changed from proximity to green space to victims of harassment - |
| **Target 11.a:** Prepare and implement a national­ urban and human settlements policy framework.  **Indicator 11.a:** Presence of a national urban and human settlements policy framework.  **Target 11.b.** By 2020, increase by x% the number of cities and human settlements adopting and implementing integrated policies and plans towards inclusion, resource efficiency, mitigation and adaptation to climate change, resilience to disasters, develop and implement in line with the forthcoming Hyogo Framework holistic disaster risk management at all levels.  **Indicator 11.b:** Covered under Indicator 11.5 above.  **Target 11.c:** Support national, regional and local governments through financial and technical assistance to strengthen revenue streams, regulatory and institutional capacity  **Indicator 11.c**: Sub-national government revenues and expenditures as a percentage of general government revenues and expenditures | 11.a Support positive economic, social and environmental links between urban, peri-urban and rural areas by strengthening national and regional development planning  11.b By 2020, substantially increase the number of cities and human settlements adopting and implementing integrated policies and plans towards inclusion, resource efficiency, mitigation and adaptation to climate change, resilience to disasters, and develop and implement, in line with the Sendai Framework for Disaster Risk Reduction 2015–2030, holistic disaster risk management at all levels  11.c Support least developed countries, including through financial and technical assistance, in building sustainable and resilient buildings utilizing local materials | 11.a.1 Proportion of population living in cities that implement urban and regional development plans integrating population projections and resource needs, by size of city  11.b.1 Proportion of local governments that adopt and implement local disaster risk reduction strategies in line with the Sendai Framework for Disaster Risk Reduction 2015-2030*  11.b.2 Number of countries with national and local disaster risk reduction strategies*  11.c.1 Proportion of financial support to the least developed countries that is allocated to the construction and retrofitting of sustainable, resilient and resource-efficient buildings utilizing local materials | **Target 11.a** – made more proactive in support of multi-sectoral links and formulation of appropriate policies  **Indicator 11.a.1** reformulated from presence of a policy to the proportion of population living in cities with appropriate plans  **Target 11.b.i** – changed from being same as Target 11.5 **to** a ‘substantial ‘increase in cities adopting and implementing such policies; Hyogo Framework updated to Sendai Framework adopted later in 2015.  **Indicator 11.b** – newly added  Target 11.c. refocused from all to developing countries, and from general institutional and capacity strengthening to sustainable and resilient construction of sustainable and resilient buildings with local materials  **Indicator 11.c.1** – major refocusing from sub-national governmental revenues /expenditures as percentage of general government revenues/expenditures to the proportion of support rather than just support, and on aid only to least developed countries instead of just support to such countries |

Source: Authors’ compilation
